# Major SCP/TAPS protein expansion in *Lucilia cuprina* is associated with novel tandem array organisation and domain architecture

**DOI:** 10.1186/s13071-020-04476-6

**Published:** 2020-11-27

**Authors:** Yair D. J. Prawer, Andreas J. Stroehlein, Neil D. Young, Shilpa Kapoor, Ross S. Hall, Razi Ghazali, Phillip Batterham, Robin B. Gasser, Trent Perry, Clare A. Anstead

**Affiliations:** 1 Bio21 Molecular Science and Biotechnology Institute, The University of Melbourne, Parkville, VIC 3010 Australia; 2 Department of Veterinary Biosciences, Melbourne Veterinary School, The University of Melbourne, Parkville, VIC 3010 Australia

**Keywords:** *Lucilia cuprina*, SCP/TAPS protein, CAP superfamily, Host-parasite interactions, Fly biology

## Abstract

**Background:**

Larvae of the Australian sheep blowfly, *Lucilia cuprina*, parasitise sheep by feeding on skin excretions, dermal tissue and blood, causing severe damage known as flystrike or myiasis. Recent advances in -omic technologies and bioinformatic data analyses have led to a greater understanding of blowfly biology and should allow the identification of protein families involved in host-parasite interactions and disease. Current literature suggests that proteins of the SCP (Sperm-Coating Protein)/TAPS (Tpx-1/Ag5/PR-1/Sc7) (SCP/TAPS) superfamily play key roles in immune modulation, cross-talk between parasite and host as well as developmental and reproductive processes in parasites.

**Methods:**

Here, we employed a bioinformatics workflow to curate the SCP/TAPS protein gene family in *L. cuprina*. Protein sequence, the presence and number of conserved CAP-domains and phylogeny were used to group identified SCP/TAPS proteins; these were compared to those found in *Drosophila melanogaster* to make functional predictions*.* In addition, transcription levels of SCP/TAPS protein-encoding genes were explored in different developmental stages.

**Results:**

A total of 27 genes were identified as belonging to the SCP/TAPS gene family: encoding 26 single-domain proteins each with a single CAP domain and a solitary double-domain protein containing two conserved cysteine-rich secretory protein/antigen 5/pathogenesis related-1 (CAP) domains. Surprisingly, 16 SCP/TAPS predicted proteins formed an extended tandem array spanning a 53 kb region of one genomic region, which was confirmed by MinION long-read sequencing. RNA-seq data indicated that these 16 genes are highly transcribed in all developmental stages (excluding the embryo).

**Conclusions:**

Future work should assess the potential of selected SCP/TAPS proteins as novel targets for the control of *L. cuprina* and related parasitic flies of major socioeconomic importance.
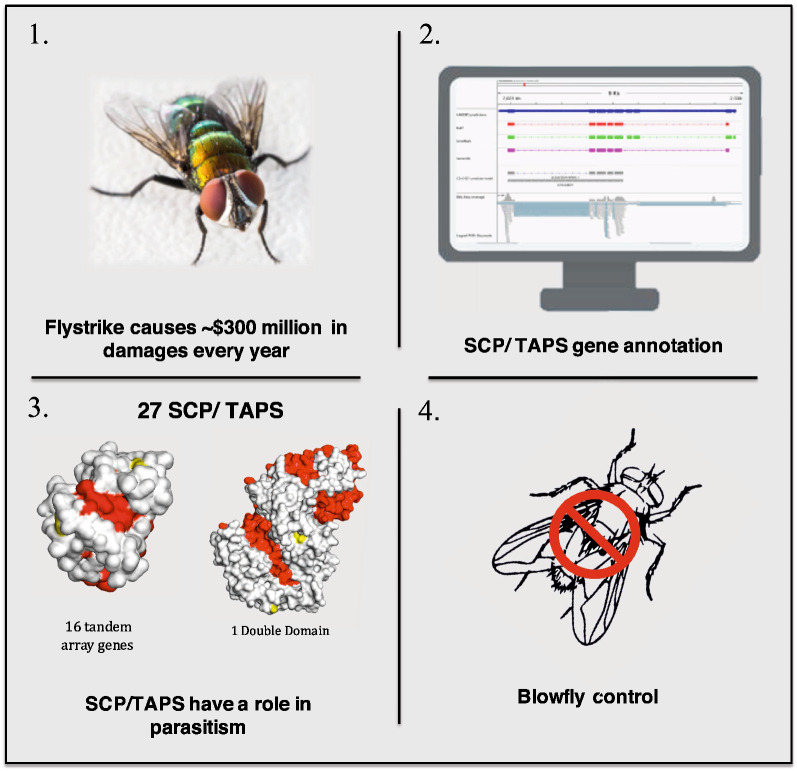

## Background

Flystrike (cutaneous myiasis), caused by the Australian sheep blowfly, *Lucilia cuprina*, is a disease of major economic significance in livestock animals worldwide [1–6]. Traditionally, the control of this disease has relied heavily on insecticide use, management procedures (e.g., surgical mulesing) or a combination of these measures [7]. However, due to resistance against most insecticides in current use [8–10] and pressure from animal welfare groups to eliminate surgical mulesing [11], there is a need for an alternative approach to treatment or control. A possible pathway to new intervention strategies could be to interfere with biological processes or pathways in *L. cuprina* and/or disrupting the parasitic relationship between the fly and its host.

Available genomic resources for *L. cuprina* [12] provide an excellent opportunity to explore biological processes and pathways in the fly as well as host-fly interactions and the pathogenesis of flystrike at the molecular level. Indeed, previous work [12] eluded to a number of groups of molecules, such as GTPases, gustatory and odorant receptors and serine proteases, some of which could be involved in parasitism (the fly-host interplay) and might be potential intervention targets. One such group is the cysteine-rich secretory protein/antigen 5/pathogenesis related-1 (CAP) or sperm-coating protein/TAPS (Tpx-1/Ag5/PR-1/Sc7) (SCP/TAP) protein superfamily, typified by a conserved CAP domain (Pfam: PF00188, InterProScan: IPR014044) [13].

Most SCP/TAPS proteins characterised to date are excretory/secretory and appear to function extracellularly [14, 15]. Many are implicated in male reproductive processes [16, 17], host-parasite interactions [18–21] and host immune modulation [22–25]. SCP/TAPS proteins are also components of venoms produced by wasps, ants and some reptiles and are present in the saliva of some blood-feeding insects [26–29]. Although the functions of many such proteins are enigmatic, some are proposed to be proteases, protease inhibitors, signaling molecules, ion channel regulators or membrane disrupters [14]. The best characterised insect, *Drosophila melanogaster* (vinegar fly), has 26 SCP/TAPS proteins (representing two groups), 15 of which are preferentially expressed in adult males and proposed to be involved in male reproduction [16]. Despite this information, almost nothing is known about SCP/TAPS proteins in *L. cuprina.* In the present study, we identified the full complement of SCP/TAPS genes and inferred proteins in *L. cuprina*, studied the structure and organisation of these genes and explored their transcription profiles in different stages/sexes of the fly as a foundation for future investigations of their functional roles and possible potential as intervention targets.

## Methods

### Annotation and 3D modelling of SCP/TAPS proteins

Total RNA was isolated separately from eggs, mixed larval stages (1st, 2nd and 3rd instars), an adult male and an adult female of *L. cuprina* and sequenced using established methods [12]. RNA-sequencing (RNA-seq) data were mapped to an enhanced version of *L. cuprina* genomic scaffolds (GenBank accession no. JRES00000000.1) using the program TopHat version 2.0.14 [30] and assembled into transcripts using Cufflinks version 2.2.1 [31], and SCP/TAPS protein-coding genes were identified using TransDecoder [32] (https://github.com/TransDecoder/TransDecoder/wiki). Transcripts with a conserved CAP domain (Pfam ID "PF00188" and/or InterProScan ID "IPR014044") were identified using InterProScan 5 [33] and aligned to genomic scaffolds using BLAT version 35.1 [34] employing the “-fine” parameter. Additionally, GeneMark version 4.2.9 [35] employing the “-ES” parameter was used to infer gene models for the eight scaffolds that contained BLAT hits. To infer gene models using Exonerate version 2.2.0 [36], the positions of BLAT hits were first extracted to restrict gene prediction to candidate gene regions and then extended up- and down-stream to predict complete, full-length gene models. These regions were analysed employing the “Coding2genome:bestfit” model. Finally, MAKER3 predictions (Anstead et al., unpublished), BLAT hits, gene predictions inferred using GeneMark and Exonerate, genomic scaffolds and RNA-seq transcripts were displayed and manually inspected in the Integrative Genomics Viewer (IGV; version 2.4.3) [37]. Manual curation of SCP/TAPS coding regions was performed by analysing each gene prediction in IGV and determining the optimal gene model based on the following criteria: (1) each gene model had to have an open reading frame (ORF) with unambiguous intron-exon boundaries supported by RNA-seq expression data; (2) coding regions could not be smaller than 400 bp, in accordance with the minimal length of the CAP domain (IPRO14044; 150 amino acids); (3) a minimum of five RNA-seq reads had to be mapped to the coding region. Alternative transcripts were not included in this analysis. Three dimensional (3D) structures of individual curated amino acid sequences were modelled using the program I-TASSER [38] and displayed using PyMOL (The PyMOL Molecular Graphics System, version 1.2r3pre, Schrödinger, LLC.)

### Confirmation of scaffold assembly

The assembly of selected scaffolds was confirmed by mapping data from 5 kb and 3 kb mate pair libraries and 100 bp paired-end libraries using Bowtie2 v.2.1.0 [39], and read-mapping coverage was calculated using BEDtools [40] and deepTools [41]. Next, high-molecular-weight genomic DNA (gDNA) was extracted from one adult male *L. cuprina*, which was digested in CTAB buffer containing proteinase K (20 mg/ml, Clontech, Hilden, Germany) and incubated for 24 h at 37 °C, followed by standard phenol/chloroform extraction [42]. Samples were not vortexed, and wide-bore pipette tips were used to reduce the chance of shearing DNA. Genomic DNA was sequenced using the MinION Mk1B SQK-LSK108 1D gDNA kit for long reads (> 100 kb) without size selection. Base calling was performed using Albacore version 2.2.7 (Oxford Nanopore) (https://github.com/zhaoc1/nanoflow) within the MinKNOW software. Reads that passed base calling quality control were aligned to genomic scaffolds using GraphMap [43] and Minimap2 [44], and the consensus sequences were analysed using NCBI motif finders [45] to identify CAP domains. Intronic regions of all 16 CAP genes located on the array were analysed for duplicated intronic sequences using BLAST [46] and MUSCLE [47] to confirm the integrity of the scaffold assembly. Finally, ten arthropod genomes (*Aedes aegypti, Ae. albopictus, Anopheles albimanus, Glossina austeni, G. brevipalpis, G. fuscipes, G. pallidipes, G. palpalis, Musca domestica* and *Stomoxys calcitrans*) were obtained from VectorBase (https://vectorbase.org/) and the *Drosophila melanogaster* genome from FlyBase [48] and were interrogated to identify SCP/TAPS tandem array genes. All listed genes containing the CAP domain (IPR014044) were downloaded (7 March 2019) and gene position data were analysed to determine tandem array SCP/TAPS proteins.

### Transcription profiling

The transcription profiles for SCP/TAPS genes were determined using RSEM [49] employing stringent criteria. Data from paired-end RNA-seq libraries (i.e., female, male and mixed larvae) were mapped using TopHat, employing stringent settings of 100% identity and not allowing multi-mapping and insertion/deletion events, but permitting gaps. These conditions were used to ensure accurate measurements of gene expression as mapping using standard parameters can make it difficult to distinguish between highly similar genes. RSEM transcription levels were normalised and represented as TPM (transcripts per million) per gene across the three RNA-seq libraries.

### Phylogenetic analysis of SCP/TAPS proteins

Phylogenetic analysis of *D. melanogaster* [48] and *L. cuprina* SCP/TAPS amino acid sequence data was performed using Geneious v.7.1.9 [50]. SCP/TAPS amino acid sequences were aligned using MUSCLE [47]. Alignment positions representing a gap in more than 50% of all aligned sequenced were manually removed. Bayesian inference trees were built using the program MrBayes v.3.2.2 [51] and rooted using a *Caenorhabditis elegans* SCP/TAPS gene (NP_001256323.1) as an outgroup. MrBayes parameters were set, as described previously [52]. Trees were displayed and modified using the programs FigTree v.1.4.1 (https://tree.bio.ed.ac.uk/software/figtree/) and Inkscape (https://www.inkscape.org/en/).

### Molecular amplification of selected SCP/TAPS genes

Total RNA was extracted from a laboratory strain (LS) of *L. cuprina* (provided by Dr Peter James, University of Queensland, Australia) from all life stages (i.e., egg, all three larval stages and both male and female adults). Total RNA was extracted using the TRIsure RNA extraction protocol (Bioline Pty Ltd, New South Wales, Australia) according to the manufacturer’s instructions and reverse transcription was performed using 2 μg of total RNA per sample using GoScript Reverse Transcription System (Promega). Primers (Additional file 1: Table S1) were designed manually or using the program Geneious v.7.1.9 and 3’ and 5’ RACE protocols (Clontech Laboratories Inc., Palo Alto, CA, USA) were employed in accordance with the manufacturer’s directions to amplify selected proteins.

## Results

### *Lucilia cuprina* has an expanded SCP/TAPS gene set representing three distinct groups

**Fig. 1:**
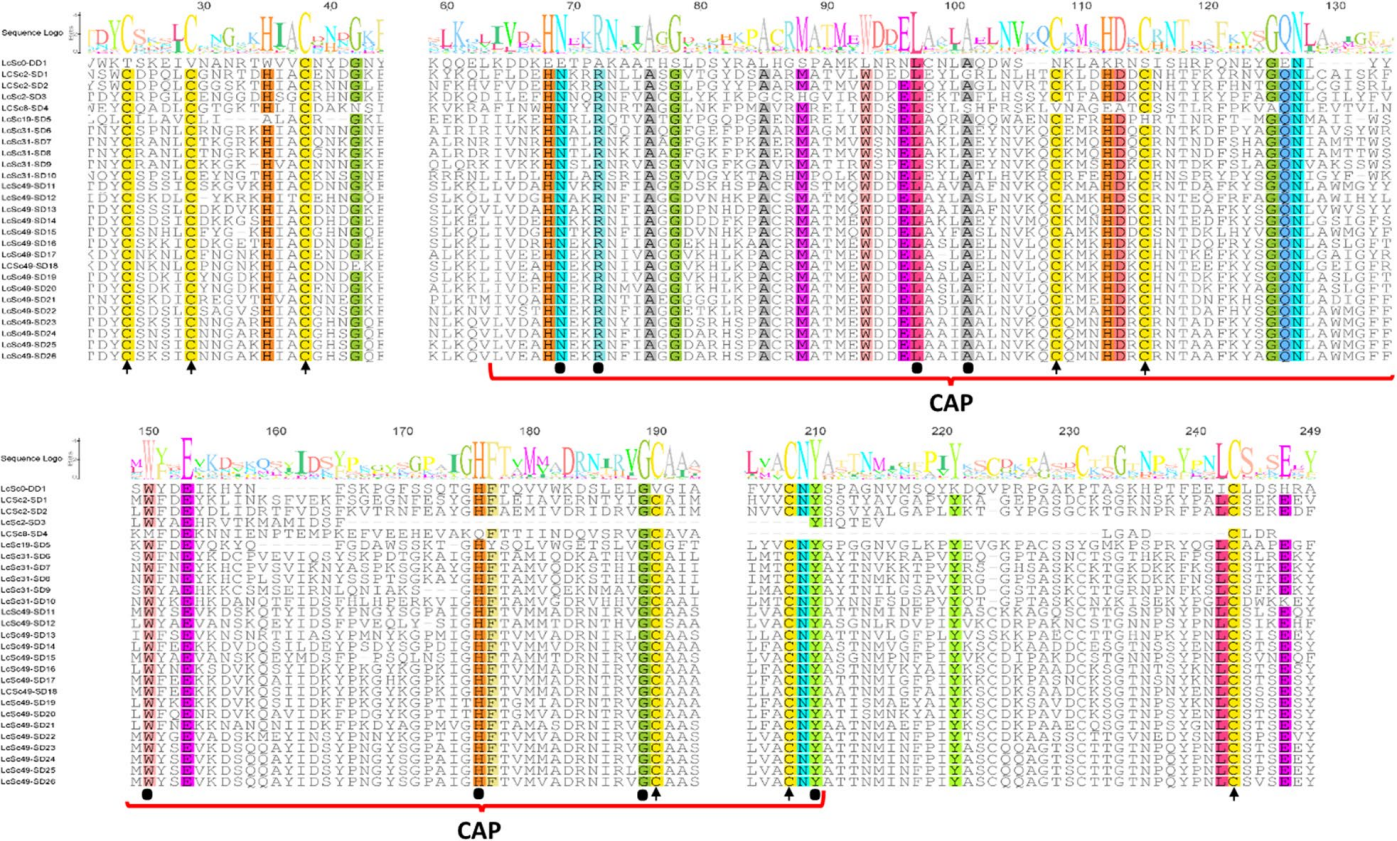
Alignment of 27 *L. cuprina* SCP/TAPS. The 27 SCP/TAPS identified in *L. cuprina* were aligned using MUSCLE (47) and manually filtered to remove alignment positions with > 50% gap frequency. Conserved cysteine residues are highlighted in yellow and indicated by an arrow. Black dots represent conserved residues common to the CAP domain. Red bars below the sequence alignment indicate CAP domain location. The sequence logo indicates the prevalence of an amino acid at each position based on the size of the letter, and numbers indicate the amino acid position in the alignment. Residues that are highly conserved (≥ 90%) are highlighted based on biochemical properties (see Geneious version 7.1.9). Regions with low (< 30%) similarity (such as the proximal CAP domain of LCSc0-DD1) are not displayed here

A total of 75 transcripts containing a conserved CAP domain were identified and mapped to six genomic scaffolds (Additional file 2: Data S1). Based on these mapped regions, 27 genes belonging to the SCP/TAPS family were predicted (Additional file 3: Data S2). Curation of the inferred gene models identified one gene coding for a solitary double-domain (DD) protein (denoted as LCSc0-DD1) with two conserved CAP domains and 26 genes coding for single-domain (SD) proteins with a solitary CAP domain (Additional file 4: Data S3). The majority (81%) of these SD proteins displayed a moderate to high degree of amino acid similarity within the CAP domain (65–100%), whereas the DD protein displayed moderate (*x̄* = 47%) sequence similarity to the SD proteins (Fig. 1). The proximal CAP domain aligned poorly with SD proteins (below the 30% threshold) and is therefore not displayed in Fig. 1. All 26 SD proteins contained conserved cysteine residues critical for the formation of disulphide bonds, a feature common to those proteins belonging to the SCP/TAPS family [15]. The majority (approximately 65%) had cysteine residues located in conserved positions (C1–C2 between 20 and 30, C3–C4 between 106–117 and C5–C6 between 224 and 233), while the remaining proteins displayed a greater variety in cysteine residue position.

Phylogenetic analyses of the 27 SCP/TAPS proteins suggested three main clades: those encoded by genes on scaffold xfSc0000049, those on scaffold Sc0000031 and those on scaffold Sc0000002 (Fig. 4). SD protein LCSc8-SD4 did not group with any other SD proteins, while DD protein LCSc0-DD1 did not cluster with any of the 26 SD proteins (Fig. 4). Phylogenetic analyses comparing SCP/TAPS proteins found in *D. melanogaster* and *L. cuprina* demonstrated that the 16 tandem array genes formed a clade with 2 *D. melanogaster* proteins *Agr* and *Agr2* (Fig. 4), and syntenic analysis of the associated genes revealed the presence of the same gene (*rutabaga*; FlyBase ID: FBgn0003301) upstream of both the 16 tandem array genes in *L. cuprina* and the *Agr* and *Agr2* genes in *D. melanogaster*. Furthermore, three *L. cuprina* genes had single-copy orthologs in *D. melanogaster* (i.e., LCSc8-SD4 to CG43775, LCSc19-SD5 to CG8483 and LCSc31-SD10 to CG9400).

### Most SCP/TAPS protein-encoding genes are organised in a large tandem array

**Fig. 2:**
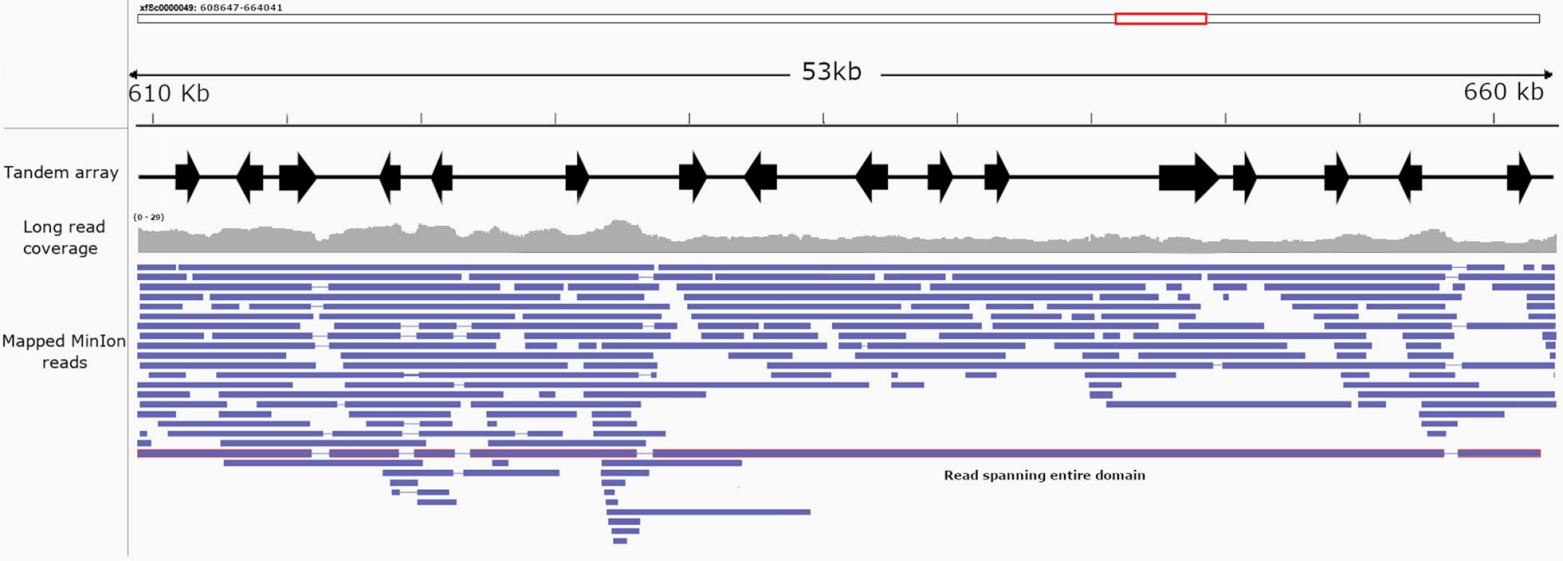
Representation of the SCP/TAPS tandem array in *L. cuprina.* A 53 kb region of scaffold xfSc0000049 containing a tandem array of 16 SCP/TAPS genes. Genes (black arrows with transcription orientation indicated by arrow), Oxford Nanopore MinION mapped long reads (blue) with associated coverage plot (grey) are pictured. A mapped long read is shown depicted with a red outline highlighting that a single read spans the entire tandem array

Sixteen SCP/TAPS genes of *L. cuprina* formed a tandem array of 53 kb on scaffold xfSc0000049 (Fig. 2), with all the encoded proteins (*n* = 16) displaying a moderate to high degree (60–95%) of amino acid similarity upon pairwise comparison. Additionally, the tandem array proteins possessed a unique genomic architecture in that each of the 16 transcripts encoded ~250 amino acid residues spanning three similarly sized exons and intronic domains of similar length, except for LCSc49-SD13 and LCSc49-SD22 (Additional file 3: Data S2). The CAP domain was conserved among proteins encoded by these 16 genes relative to other *L. cuprina* SCP/TAPS proteins exhibiting 76%–100% amino acid similarity upon pairwise comparison (Fig. 1). All SCP/TAPS in this array contained the CRISP motif [14] with up to ten conserved cysteine residues required for disulphide bond formation [15]. These residues were positioned in similar locations along the transcript, which is consistent with the location of these residues found in other species [53]. Alignment of *L. cuprina* RNA-seq libraries (i.e., female, male and mixed larvae) indicated that each gene was transcribed. Stringent mapping of transcriptomic reads to the entire scaffold containing the putative tandem array demonstrated that each gene was supported by RNA-seq data. All genes had at least ten RNA-seq reads mapped across the entire coding domain. Predicted protein models verified the core structural elements of the SCP/TAPS proteins (i.e., the α-β-α sandwich and stabilizing disulphide bonds). These structural elements are largely comprised of conserved CAP residues and appear to be internal while variable sequence regions more commonly coincide with amino acids located on the protein surface (Additional file 5: Figure S1, Additional file 6: Data S4).

When the data from the 5 kb and 3 kb mate-pair libraries and 100 bp paired-end reads (SUB3252906) were mapped to scaffold xfSc0000049, consistent coverage was observed across predicted coding regions. Long-read sequencing data using MinION sequencing (Oxford Nanopore Technologies, Oxford, UK) and subsequent read-mapping supported the integrity of scaffold xfSc0000049. One 61,501 bp read (ID: 102324e2-a088-4652-a4d2-7164e8fb4f52) mapped across the entire scaffold, providing additional support for this region (Fig. 2). Coverage analysis using the software package plotCoverage revealed a mean coverage of 12.4 reads and 80% of all positions had a coverage of greater than 9 reads across the scaffold xfSc0000049 region (xfSc0000049: 608647-664041). In addition, CIGAR string extraction showed 84.7% of all mapped nucleotide positions were matches. The consensus sequence shared 96.1% sequence identity with the reference scaffold and all 16 CAP domains were present in the consensuses sequences when analysed using NCBI motif finders. Finally, the analysis of 11 related arthropod genomes did not identify SCP/TAPS genes arranged in a tandem array to the extent identified in this study (Additional file 7: Table S2).

### A novel double-domain CAP protein identified in *L. cuprina*

**Fig. 3:**
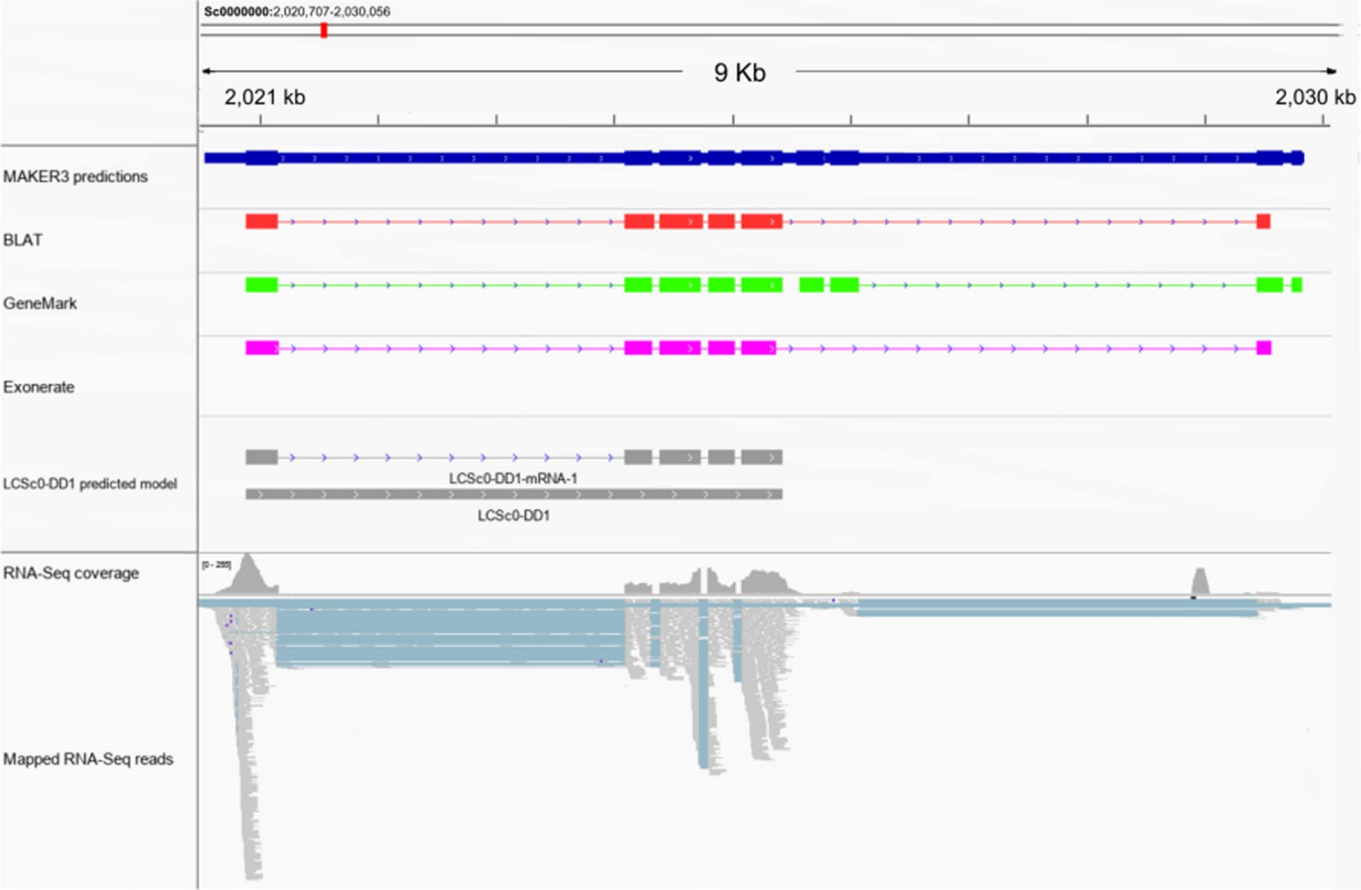
Representation of LCSc0-DD1 protein in *L. cuprina* genomic scaffolds. A predicted gene model (grey) consisting of five exons encoding a double-domain protein (LCSc0-DD1); supported by complementary data from the *L. cuprina* genome assembly, MAKER 3 predictions (blue), BLAT transcript mapping (red), gene prediction tools GeneMark and Exonerate (green and pink, respectively) and RNA-seq data from all life stages with associated coverage plot. Raw files supplied in Additional file 8: Data S5

One of the 27 SCP/TAPS protein genes inferred here encoded a unique double-domain protein (LCSc0-DD1). Two independent gene prediction tools (i.e., GeneMark and Exonerate) inferred slightly different models for this unique gene. Of these models, only five exons were supported by RNA-seq data, suggesting the predicted gene model inferred by GeneMark was accurate (Additional file 8: Data S5). This gene model was curated manually to ensure that the appropriate splice junctions and open reading frame across the five predicted exons was evident. This model was fully supported by the MAKER3 prediction (Anstead 2020, unpublished) and BLAT-mapped transcripts. Exonerate predictions also supported the curated model; however, a putative downstream exon was also predicted in both cases (Fig. 3, Additional file 8: Data S5).

Predicted gene models were verified employing conventional PCR strategies using primers flanking the conserved CAP domains. Transcripts containing both CAP domains were successfully amplified from a male cDNA library; however, a complete transcript was not amplified using conventional and/or RACE amplification strategies. The presence of two CAP domains in a single cDNA amplicon provides strong support for the presence of a double-domain protein in *L. cuprina* (Additional file 9: Data S6).

### Diverse transcription profiles for SCP/TAPS protein-encoding genes

**Fig. 4:**
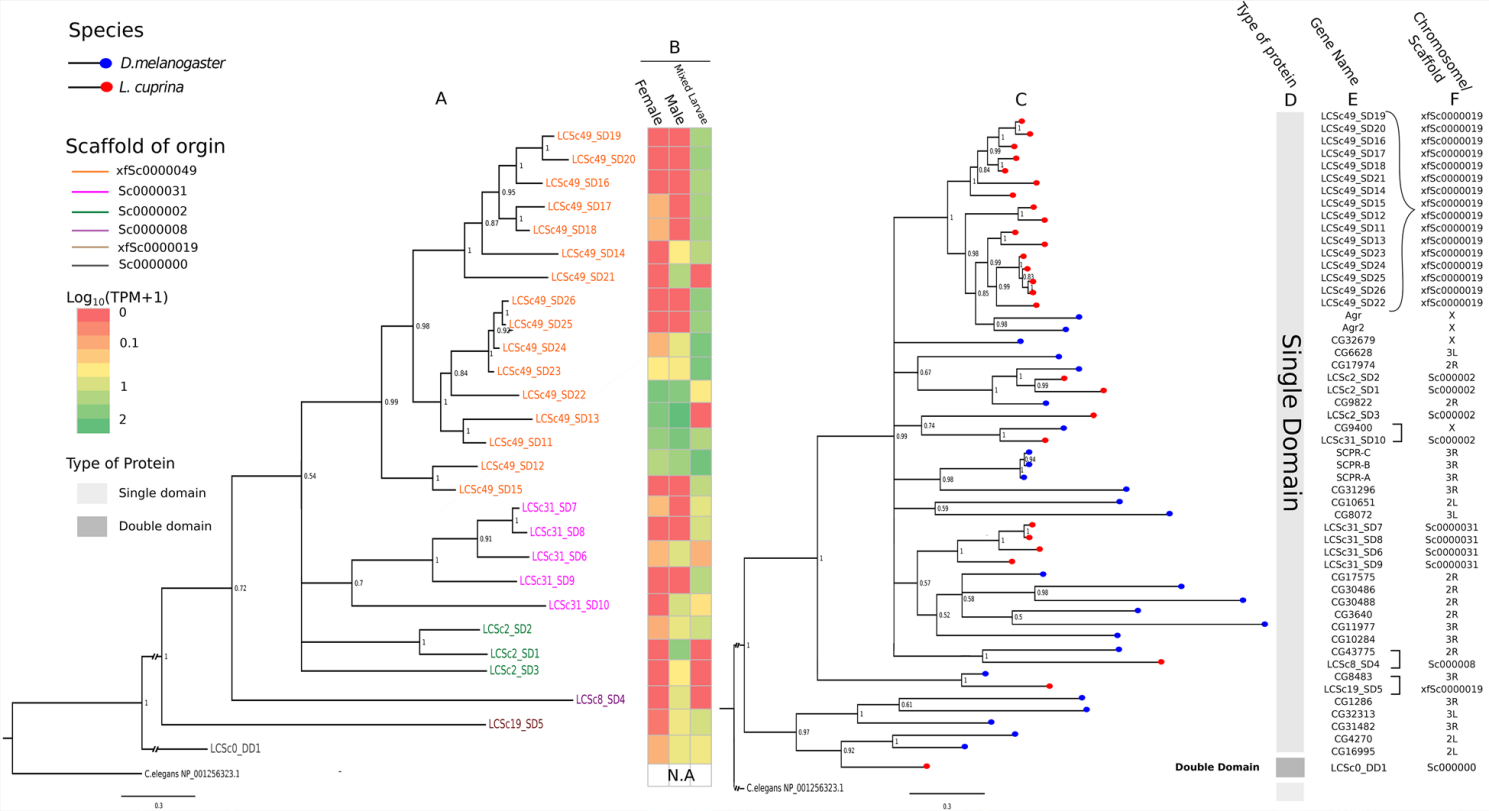
Phylogenetic relationships among curated SCP/TAPS proteins. **a** Phylogenetic tree of 27 SCP/TAPS predicted protein sequences derived from *L. cuprina*. Colours indicate the scaffold from which the SCP/TAPS gene originated. **b** Heatmap displaying log_10_(TPM+1)-scaled transcription levels for each SCP/TAPS gene across the adult female, adult male and mixed larval libraries. Unadjusted TPM values can be obtained from Additional file 10: Table S3. **c** Phylogenetic tree of SCP/TAPS in both *L. cuprina* (red) and *D. melanogaster* (blue). **d** Indicates a Double Domain or Single Domain protein and **e** gene name of SCP/TAPS proteins from both species in the order in which they cluster. The curly bracket indicates genes that form the predicted tandem array and the square brackets show single-copy orthologs and **f** the encoding scaffold or chromosome. Trees were rooted using the *C. elegans* SCP/TAPS protein NP_001256323.1 as an outgroup and constructed using Bayesian inference (MrBayes v3.3.3) with branch labels indicating posterior probability values

Although transcription profiles for the individual SCP/TAPS genes varied among libraries (Additional file 10: Table S3), all genes were transcribed in at least one of the RNA-seq libraries (TPM: 0.4 to 456.8). Eleven of the 16 genes (i.e., LCSc49-SD14, LCSc49-SD15, LCSc49-SD16, LCSc49-SD17, LCSc49-SD18, LCSc49-SD19, LCSc49-SD20, LCSc49-SD23, LCSc49-SD24, LCSc49-SD25 and LCSc49-SD26) in the tandem array were predominantly transcribed in mixed-larval samples, whereas two tandem-array genes (LCSc49-SD13 and LCSc49-SD22) were expressed in male and female RNA-seq libraries. Two genes (LCSc49-SD11 and LCSc49-SD12) in the tandem array were transcribed in all three RNA-seq libraries, and one gene (LCSc49-SD21) was specifically transcribed in the library representing adult males (Fig. 4). The TPM (transcripts per million) values for genes in the tandem array (Additional file 10: Table S3) were significantly higher (*P* < 0.05) (mean: 46.6) compared with all other SCP/TAPS genes (mean: 4.3), indicating higher transcription levels for genes within the tandem array relative to other SCP/TAPS protein genes in *L. cuprina.* Additionally, two genes (i.e., LCSc2-SD1 and LCSc31-SD9) that were not encoded in the array had TPM values > 10 (Additional file 10: Table S3). Furthermore, LCSc0-DD1 also displayed low levels of transcription across the three libraries (female: 0.2, male: 1.88, mixed larvae: 1.34) indicating that the double-domain protein was predominantly transcribed in adult male and mixed larval samples, albeit at low levels (Fig. 4).

## Discussion

Here, we annotated and curated all SCP/TAPS protein-encoding genes in *L. cuprina*, classified them using a phylogenetic approach, established their genomic locations and organisation, assessed the transcription profiles of individual genes and explored the domain architectures of encoded proteins.

SCP/TAPS proteins share characteristic structural components that typify this protein superfamily [13]. One such feature is the α-β-α sandwich structure, which usually comprises ~50% of the protein structure [21] and is conserved among species. The variability in the remaining 50% of the protein structure suggests that the CAP domain forms a versatile molecular framework, enabling the diversification of SCP/TAPS protein function [21]. This proposal of functional diversification is supported, to some extent, by marked variation in transcription profiles between adult and larval stages and sexes of the fly; in adult flies, some of these molecules might associate with basic reproductive and developmental processes, as shown in *Drosophila*, whereas in larvae, others might assume roles in regulating protease activity, protease inhibition and/or a modulation of the host immune responses during larvae establishment and parasitism [19, 20]. Clearly, these are exciting areas worthy of pursuit using a combination of technological approaches, including immunoproteomics, transcriptomics and in vivo experiments in sheep.

*Lucilia cuprina* tandem array genes (TAGs) were variable, with predicted models suggesting that variable sequence regions (non-CAP residues) comprise much of the protein surface, while conserved residues form the internal protein structure (Additional file 5: Figure S1). It would be tempting to suggest that these proteins are under selective pressure, but future work is required to assess dN/dS ratios linked to tandem arrays of SCP/TAPS protein and other (e.g., yolk protein) genes [54] in *L. cuprina* and other flies to explore the evolutionary mechanisms leading to and maintaining these arrays. The solitary double-domain SCP/TAPS protein (LCSc0-DD1) characterised here is of particular interest; like solitary CAP domain proteins, double-domain proteins have also been associated with many crucial parasitic functions [15, 24, 55–60]. Given this association in other parasitic organisms, the presence of a double-domain protein in the *L. cuprina* genome warrants further investigation of the suspected role of LCSc0-DD1 in parasitism and whether LCSc0-DD1 could be a suitable vaccine target.

Analyses of the SCP/TAPS gene family in *L. cuprina* identified an extended tandem array of 16 SCP/TAPS genes located in a 53 kb domain of scaffold xfSc0000049. Although SCP/TAPS protein genes are represented in triplets in the genome of *D. melanogaster* [16] or aggregate as a series of four genes in the genome of the blood fluke *Schistosoma mansoni* [15], the tandem array identified here spans 16 genes and is significantly larger than aggregates seen in known SCP/TAPS tandem arrays. This appears to be unique to *L. cuprina*, as our analysis of 11 related arthropod genomes did not identify similar TAGs of the extent seen here (Additional file 7: Table S2). It is important to note that this analysis is preliminary, and future work should investigate a wider array of genomes in greater detail to establish whether or not this array is unique to *L. cuprina*. Nevertheless, our data suggest a large-scale gene duplication or replication event, resulting in a number of very similar and structurally conserved proteins forming this tandem array. An analysis of this region employing long-read sequencing data and RNA-seq data provided convincing evidence for the presence and transcription of 16 separate genes containing the SCP/TAPS domain. Tandem duplication events have been shown to drive the development and maintenance of gene superfamilies [61] and are a common mechanism used to adapt to harsh environments [62–65]. Alternatively, tandem array proteins may be a mechanism to promote production of large amounts of protein product, representing an alternative to other means of gene upregulation [66]. Given that *L. cuprina* TAGs are predominantly transcribed in the parasitic life stages (i.e., larval stages) and are transcribed at higher levels relative to other SCP/TAPS protein genes of *L. cuprina*, it is plausible that large-scale tandem duplications of these genes are a means by which *L. cuprina* has upregulated the production of these proteins. As SCP/TAPS proteins are commonly detected in excretory/secretory products [67] and proposed to modulate host immune responses [21], it is plausible that *L. cuprina* transcribes and expresses some that play a key role in host-larval interactions, reflected in their upregulation during parasitism. Investigating the cross-talk between fly larvae and the host animal could provide clues as to how to disrupt this relationship and might identify novel intervention targets for flystrike.

TAG function may also be linked to digestion as *D. melanogaster* proteins (*Agr* and *Agr2*) cluster phylogenetically with the 16 SCP/TAPS gene tandem array in *L. cuprina* and are known to be highly upregulated in digestive tissues during larval stages [48, 68, 69]. Synteny analysis of the associated genes revealed the presence of the same gene (*rutabaga*) upstream of both the *L. cuprina* tandem array and the *Agr* and *Agr2* genes in *D. melanogaster*, suggesting a close evolutionary relationship between the encoded proteins. *Agr* is highly expressed in larval salivary glands, while *Agr2* is predominately expressed in larval hindgut and midgut, signifying a role in larval digestion [48, 69]. The need for *L. cuprina* larvae to rapidly and effectively digest tissue could explain the necessity for a large-scale duplication/replication event resulting in a tandem array. Interestingly, transcription profiles for SCP/TAPS protein genes in *L. cuprina* were distinct concordant with those of *D. melanogaster*, suggesting that at least some of those transcribed and expressed in larval stages are required for a parasitic existence.

Despite advances in knowledge of the structural biology of CAP domains [20], their functions remain unclear, yet cysteine-rich secretory proteins have previously been the subject of successful vaccine candidate identification in several species of hookworm [70–72]*.* Hookworm L3 larvae are known to secrete two main CAP-like proteins, namely *Ancylostoma *secreted proteins 1 and 2 (i.e., ASP-1 and ASP-2) with two and one CAP domains, respectively [73]. Previous work showed that hamsters vaccinated with a recombinant form of Ac-ASP-2 exhibited a reduction in *Ancylostoma ceylanicum* burdens compared with control hamsters, while the full-length Ac-ASP-1 did not [71]. Intriguingly, immunisation with CAP domain-truncated form of Ac-ASP-1 was effective at reducing burdens compared with the full-length Ac-ASP-1, which did not [74–76], suggesting that the CAP domain and oligomerisation of the double-domain unit play a part in immunogenicity in the host animal. It is important to note that the inability to amplify a complete LCSc0-DD1 transcript using conventional and RACE PCR protocols could be in part due to the low transcription levels of this isoform or due to suboptimal primer design given the low GC content in the *L. cuprina* genome. Assessing the presence of other putative isoforms derived from LCSc0-DD1 should be the subject of further investigations as this might aid in elucidating the variability of double-domain structural isoforms and enhance our understanding of single- and double-CAP domain differences.

## Conclusions

In conclusion, future investigations of the molecular processes underlying the biological functions of these intriguing proteins should be facilitated by advances in proteomic and transcriptomic data analyses as well as continuous improvement in genome sequencing and analyses. In this context, elucidating the biological function of SCP/TAPS through the use of functional genomics tools such as the CRISPR-cas9 genome-editing system [77] would be a logical next step. Initial success in full knockout of blowfly genes has been achieved [78], supporting the validity of such an approach. Establishing such a knockout system for *L. cuprina* could enable functional assessments of SCP/TAPS proteins as vaccine or insecticide targets providing prospects toward the development of new interventions against flystrike.

## Supplementary information

**Additional file 1: Table S1.** Primers used to verify the LCSc0-DD1 protein.

**Additional file 2: Data S1.** Subset of all *L. cuprina* genomic scaffolds that contain mapped CAP transcripts.

**Additional file 3: Data S2.** GFF file for all 27 SCP/TAPS protein genes.

**Additional file 4: Data S3.** List of SCP/TAPS proteins predicted for *L. cuprina* with gene identifiers, scaffold location and predicted amino acid sequences. Gene identifiers are composed of the species of origin (LC: *L. cuprina*), scaffold number (Sc0: Scaffold Sc0000000) and whether the amino acid sequence produces a single or double-domain protein (SD or DD)

**Additional file 5: Figure S1.** A protein model displaying the surface of LCSc49-SD12 and indicating the surface of the protein for the conserved CAP domain (red), for non-CAP domain residues (white), and cysteine residues that form disulphide bonds (yellow). Cysteine residue C3-C4 are not visible on the protein surface.

**Additional file 6: Data S4. A:***L. cuprina* SCP/TAPS best-predicted protein models in .pdb file format, produced with program I-TASSER and **B:** accompanying summary statists indicating accuracy of the predicted models. C-score is a confidence score ranging [-5,2]. TM-score and RMSD measure the similarity to the native structure. TM-score ranging [0,1] with a value >0.5 implying the model has correct topology.

**Additional file 7: Table S2.** List of arthropod genomes with their respective number of predicted SCP/TAPS proteins as well as the largest number of SCP/TAPS genes clustered together in a tandem array.

**Additional file 8: Data S5.** Output files loaded into IGV to manually determine SCP/TAPS gene models A: Mapped CAP transcripts using BLAT B: Gene prediction tool GeneMark C: Gene prediction tool Exonerate.

**Additional file 9: Data S6.** LCSc0-DD1 amplicon sequencing data. A: Representation of the largest mapped amplicon to LCSc0-DD1, indicating two distinct CAP domains (red square brackets) and primer locations (red arrows). B: and C: LCSc0-DD1 amplicon sequence data in gb file format; sequenced in forward and reverse direction, respectively.

**Additional file 10: Table S3.** Transcription levels (in transcripts per million, TPM) and read counts of SCP/TAPS genes for adult female, adult male and mixed larval libraries. Tandem array SCP/TAPS are indicated with an asterisk.

## Data Availability

The datasets supporting the conclusions of this article are included within the article and its Additional files. Any additional data are available from the corresponding author upon request. Accession codes: This Whole Genome Shotgun project has been deposited at DDBJ/ENA/GenBank under the accession JRES00000000.1 (BioProject: PRJNA419080, BioSample: SAMN08042461). The genome version described in this paper is version PPXR01000000. The RNAseq and Nanopore fastq files have been deposited in the NCBI short read archive (SRA) under the accession number PRJNA419080.

## Abbreviations

DD: Double-domain protein
LS: Lab strain
SCP/TAPS: Sperm-Coating Proteins/Tpx1/Ag5/PR-1/Sc7
SD: Single-domain protein
TPM: Transcripts per million
