# Supplementary figures and images for: Major SCP/TAPS protein expansion in Lucilia cuprina is associated with novel tandem array organisation and domain architecture
Source: Parasit Vectors. 2020 Nov 27;13:598. doi: 10.1186/s13071-020-04476-6 (PMC7694928; doi:10.1186/s13071-020-04476-6)

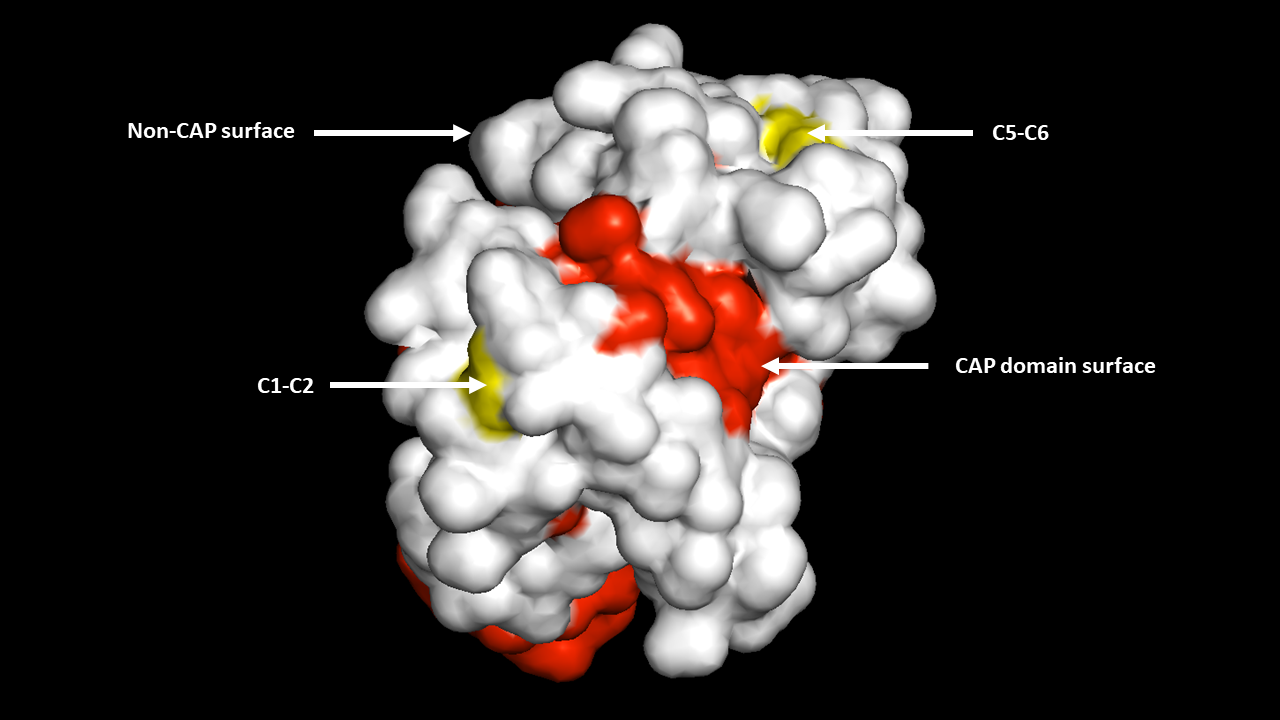

Supplement: Supplementary file 5 — Additional file 5: Figure S1. A protein model displaying the surface of LCSc49-SD12 and indicating the surface of the protein for the conserved CAP domain (red), for non-CAP domain residues (white), and cysteine residues that form disulphide bonds (yellow). Cysteine residue C3-C4 are not visible on the protein surface. [file 13071_2020_4476_MOESM5_ESM.png]

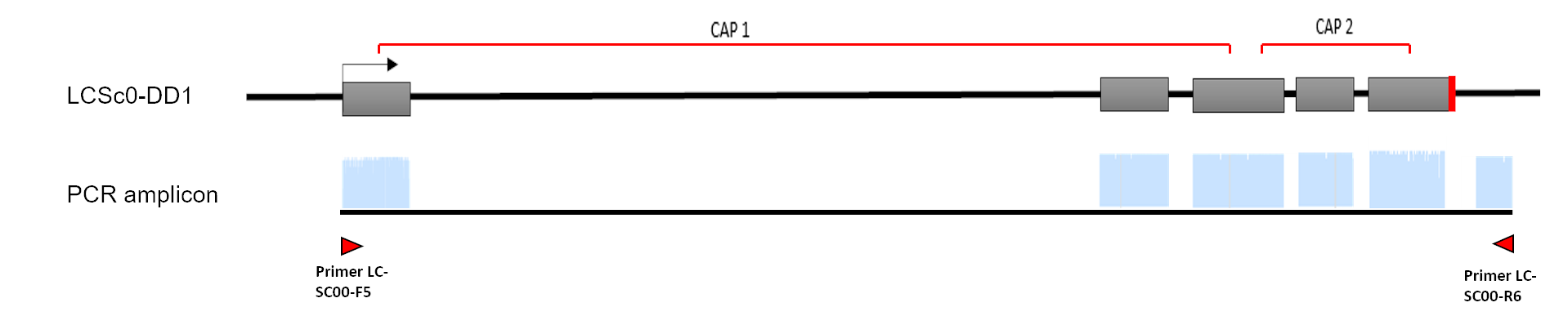

Supplement: Supplementary file 9 — Additional file 9: Data S6. LCSc0-DD1 amplicon sequencing data. A: Representation of the largest mapped amplicon to LCSc0-DD1, indicating two distinct CAP domains (red square brackets) and primer locations (red arrows). B: and C: LCSc0-DD1 amplicon sequence data in gb file format; sequenced in forward and reverse direction, respectively. [file 13071_2020_4476_MOESM9_ESM.zip › Additional file 9A.png]
